# Parasite Burden and CD36-Mediated Sequestration Are Determinants of Acute Lung Injury in an Experimental Malaria Model

**DOI:** 10.1371/journal.ppat.1000068

**Published:** 2008-05-16

**Authors:** Fiona E. Lovegrove, Sina A. Gharib, Lourdes Peña-Castillo, Samir N. Patel, John T. Ruzinski, Timothy R. Hughes, W. Conrad Liles, Kevin C. Kain

**Affiliations:** 1 Institute of Medical Science, Department of Medicine, University of Toronto, Toronto, Ontario, Canada; 2 McLaughlin-Rotman Centre for Global Health, McLaughlin Centre for Molecular Medicine, University Health Network, University of Toronto, Toronto, Ontario, Canada; 3 Department of Medicine, University of Washington, Seattle, Washington, United States of America; 4 Center for Cellular and Biomolecular Research, University of Toronto, Toronto, Ontario, Canada; 5 Department of Medical Genetics and Microbiology, University of Toronto, Toronto, Ontario, Canada; 6 Division of Infectious Diseases, Department of Medicine, University of Toronto, Toronto, Ontario, Canada; Case Western Reserve University, United States of America

## Abstract

Although acute lung injury (ALI) is a common complication of severe malaria, little is known about the underlying molecular basis of lung dysfunction. Animal models have provided powerful insights into the pathogenesis of severe malaria syndromes such as cerebral malaria (CM); however, no model of malaria-induced lung injury has been definitively established. This study used bronchoalveolar lavage (BAL), histopathology and gene expression analysis to examine the development of ALI in mice infected with *Plasmodium berghei* ANKA (PbA). BAL fluid of PbA-infected C57BL/6 mice revealed a significant increase in IgM and total protein prior to the development of CM, indicating disruption of the alveolar–capillary membrane barrier—the physiological hallmark of ALI. In contrast to sepsis-induced ALI, BAL fluid cell counts remained constant with no infiltration of neutrophils. Histopathology showed septal inflammation without cellular transmigration into the alveolar spaces. Microarray analysis of lung tissue from PbA-infected mice identified a significant up-regulation of expressed genes associated with the gene ontology categories of defense and immune response. Severity of malaria-induced ALI varied in a panel of inbred mouse strains, and development of ALI correlated with peripheral parasite burden but not CM susceptibility. *Cd36* ^−/−^ mice, which have decreased parasite lung sequestration, were relatively protected from ALI. In summary, parasite burden and CD36-mediated sequestration in the lung are primary determinants of ALI in experimental murine malaria. Furthermore, differential susceptibility of mouse strains to malaria-induced ALI and CM suggests that distinct genetic determinants may regulate susceptibility to these two important causes of malaria-associated morbidity and mortality.

## Author Summary

Acute lung injury (ALI) and acute respiratory distress syndrome (ARDS) can occur in adult malaria infections with a case fatality rate of 70%–100%. ALI and ARDS are characterized by protein-rich fluid in the lungs, with reduced gas exchange, and in malaria, often accompany high parasite levels and severe or cerebral disease. In this work we have examined lung physiology, pathology and genomics in mouse malaria—*Plasmodium berghei* ANKA—to show that mice develop malaria-induced ALI. Infected mice have proteinaceous fluid in their lungs, have a migration of inflammatory cells from the blood into the lung walls, and express immune response–related genes. We also found that severity of ALI depended on high parasite levels, both overall and specifically in the lung tissue, but was not consistent with whether the mice developed cerebral malaria. ALI due to *Plasmodium berghei* ANKA infection models prominent characteristics of human malaria-associated ALI, and we have better defined this model of malaria ALI so it may be used to further explore disease mechanisms and eventual treatment.

## Introduction

Pulmonary complications have been reported in malaria caused by infection with *Plasmodium falciparum*, *Plasmodium vivax* and *Plasmodium ovale* [1],[2]. Pulmonary edema, with features of acute lung injury (ALI) and the acute respiratory distress syndrome (ARDS), occurs in approximately 20% of severe malaria patients [3], often in association with cerebral malaria (CM), acute renal failure and high parasitemia [3],[4],[5],[6],[7],[8]. ARDS in adults is an important predictor of mortality in malaria, and is associated with a greater than 70% case fatality rate [3]. Although ALI and ARDS are rare in the pediatric population [9], respiratory distress accompanying severe metabolic acidosis is common in children and predicts poor outcome [10]. While pulmonary involvement is a recognized complication of malaria infection, little is currently known about its pathogenesis [11].

A spectrum of severity exists with respiratory involvement in malaria infection. Cough is a common presentation in uncomplicated malaria due to *P. falciparum*, *P. vivax* and *P. ovale* infections [1],[2]. Reduced gas transfer and impaired alveolar-capillary membrane function have been correlated with severe disease [2]. Patients can rapidly progress to respiratory failure, either in association with severe disease or shortly after treatment [9]. Studies suggest that this post-treatment lung injury may be associated with prolonged alveolar-capillary inflammation [1],[12]. Lung ultrastructural studies from individuals with fatal *P. falciparum*-induced lung injury indicate endothelial cell cytoplasmic swelling and edema in the lung interstitium, with monocytes and parasitized erythrocytes (PE) adherent within the capillaries [13],[14]. Additionally, septal or interstitial edema occurs in regions of PE adherence [15]. Lung endothelium likely plays an important role in malaria lung injury, in response to PE adhesion, parasite-induced inflammation (for example, by malaria GPI) and leukocyte adhesion. *In vitro*, *P. falciparum* PEs have been shown to promote oxidative stress [16], and activate caspases leading to apoptosis in human primary lung endothelial cells [16]. Both *P. falciparum* PEs and GPI induce up-regulation of endothelial inflammatory markers, including intracellular cell adhesion molecule-1 (ICAM-1; NP_000192) and interleukin-6 (IL-6; NP_000591) [17],[18],[19]. An increase in cell adhesion molecules may further enhance leukocyte and PE adhesion, contributing to localized endothelial damage.

Although the murine malaria model of *P. berghei* ANKA (PbA) has primarily been used to study CM [20], pulmonary pathology has also been described in some previously published studies that employed this model of severe malaria [20],[21],[22],[23],[24],[25],[26]. Lung histopathology of PbA-infected mice has been reported to show endothelial adhesion of pigment-containing monocytes and neutrophils, and a “septal pneumonitis” [24]. Immunoglobulins, complement 3, complement 4 and parasite antigens in the lung interstitium and alveoli were detected by immunohistochemistry one to three hours prior to death in CM-susceptible mice [22]. Studies have also demonstrated increased pulmonary vascular permeability in PbA infection [20],[23],[25], which may be influenced by CD11a-positive neutrophil and monocyte sequestration [23]. Additionally, PbA parasites sequester in lung tissue in a CD36-dependent manner [27], and the lung may be a preferential site of PbA biosynthesis and/or proliferation [28]. Collectively, these data suggest that significant lung pathology occurs in PbA infection and contributes to malaria-associated morbidity and mortality.

Since relatively little is known about lung injury in malarial disease, a mouse model could lead to pathophysiological insights with potential relevance to human disease. We hypothesized that ALI would occur in the PbA mouse model and would be mediated by parasite sequestration in the lung. Similar to severe malarial syndromes in human disease, we show that ALI develops in PbA infection, and is influenced by both parasite burden and local sequestration.

## Results

### PbA-infected mice develop ALI characterized by alveolar-capillary membrane barrier disruption

**Figure 1:**
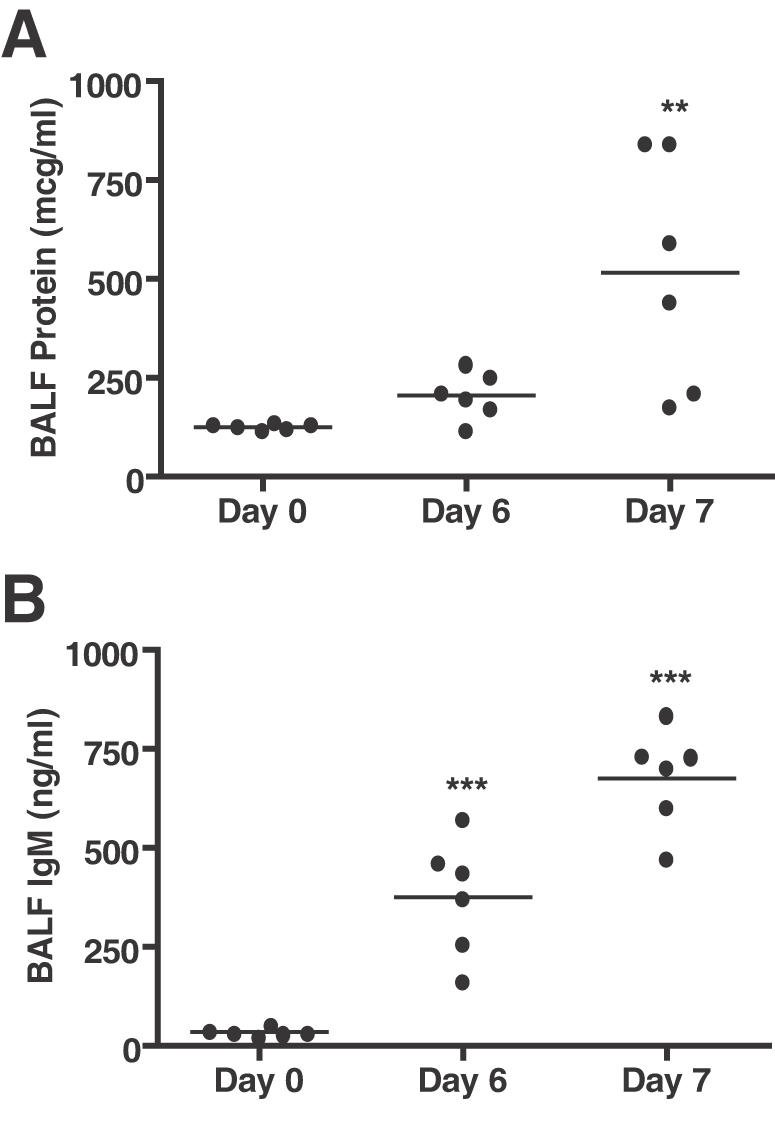
C57BL/6 Mice infected with PbA develop ALI. C57BL/6 mice infected with 1×10^6^ PbA parasites were examined for lung damage once they exhibited marked parasitemia (day 6 and 7) but before they showed cerebral symptoms (n = 6/group/experiment, representative of two independent experiments). A. Total protein concentration is significantly elevated in the BAL of PbA infected mice at Day 7 post infection (one-way ANOVA with Bonferroni's multiple comparison correction, **p<0.01). B. IgM concentrations are significantly elevated over baseline levels in the BAL fluid at both Day 6 and 7 of PbA infection (***p<0.001). Increased levels of BALF total protein and IgM are consistent with the development of pulmonary vascular leak and a disruption of the alveolar-capillary membrane that occur during ALI.

In order to characterize PbA infection as a model of malaria lung injury, bronchoalveolar lavage (BAL) was performed on C57Bl/6 mice 1–2 days prior to the development of CM symptoms and death and the BAL fluid (BALF) was examined for protein content. Increased levels of total protein, and more specifically IgM, in the BALF are indicative of alveolar-capillary membrane barrier disruption and are hallmarks of ALI [29],[30],[31]. Levels of total protein were significantly elevated at day 7 post-infection (Figure 1A, one-way ANOVA with Bonferoni's multiple comparison correction, Day 7 vs. Day 0: p<0.01). Furthermore, IgM was increased at both Day 6 and Day 7 compared to baseline (Figure 1B, p<0.001). These data showed that a disruption of the alveolar-capillary membrane barrier and ALI occur as a result of PbA infection.

### ALI caused by PbA infection is associated with increased production of pro-inflammatory cytokines in peripheral blood but not in alveolar spaces

**Figure 2:**
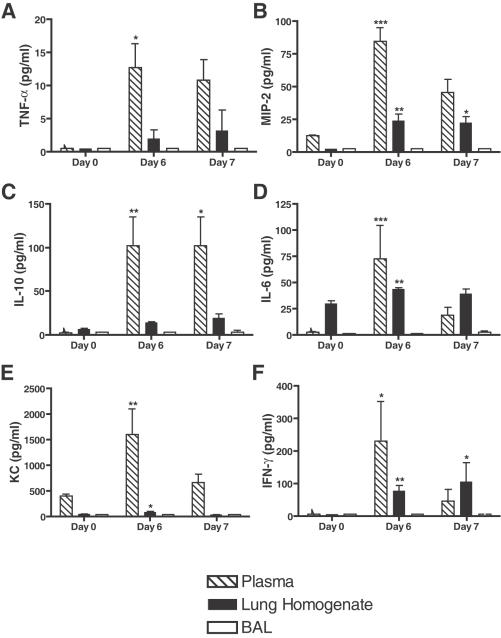
Cytokines and chemokines are not present in the BALF of PbA-infected C57BL/6 mice. A panel of cytokines and chemokines was examined in the plasma, lung homogenate and BALF of uninfected and PbA infected C57BL/6 mice (n = 6/group/experiment, representative of two independent experiments, expressed as mean +/− SEM). All cytokines/chemokines examined were elevated at D6 compared to baseline (Kruskal-Wallis test with Dunn's multiple comparison test; * p<0.05, **p<0.01, and ***p<0.001; TNF (A), MIP2 (B), IL-10 (C), IL-6 (D), KC (E) and IFN-γ (F)). IL-6 and KC were elevated in lung homogenates at day 6 versus day 0, and MIP-2 and IFN-γ levels were increased at both days 6 and 7 post-infection (Kruskal-Wallis test with Dunn's multiple comparison test; * p<0.05, **p<0.01, and ***p<0.001). TNF and IL-10 levels in lung homogenate fell below the detection range of the assay. Notably, none of the cytokines were detectable in the BALF, indicating that inflammation occurs peripherally and in the lung tissue, but not in the alveolar spaces.

To examine pulmonary inflammation induced during PbA infection, a panel of cytokines and chemokines were examined in plasma, lung tissue homogenate and BALF. PbA failed to induce proinflammatory cytokine production in the alveoli of infected mice, as measured in the BALF (Figure 2). In contrast, proinflammatory cytokine production was increased both locally (in lung tissue) and peripherally (in plasma) during the course of PbA infection. Tumor necrosis factor (TNF; NP_038721, Fig. 2A), macrophage inflammatory protein-2 (MIP-2; NP_033166, Fig. 2B), interleukin-10 (IL-10; NP_034678, Fig. 2C), IL-6 (NP_112445, Fig. 2D), keratinocyte-derived cytokine (KC or murine IL-8; NP_032202, Fig. 2E) and interferon-γ (IFN-γ; NP_032363, Fig. 2F) levels were all significantly increased in plasma at day 6 compared to baseline (Kruskal-Wallis test with Dunn's multiple comparison test; p<0.05: TNF, IFN-γ; p<0.01: IL-10, KC; p<0.001: MIP-2, IL-6). Lung homogenate levels of IL-6 and KC were significantly increased at Day 6 (p<0.01 and p<0.05, respectively) and tissue levels of MIP-2 and IFN-γ were significantly elevated at both days 6 and 7 compared to day 0 (D6 vs. D0: p<0.01; D7 vs. D0: p<0.05). Overall, both systemic and local tissue inflammation occur as a result of PbA infection, however, cytokine and chemokines are produced in the plasma and lung interstitium rather than in the alveolar spaces.

### Lung tissue of PbA-infected mice shows interstitial inflammation but no cellular infiltrates in the alveoli

**Figure 3:**
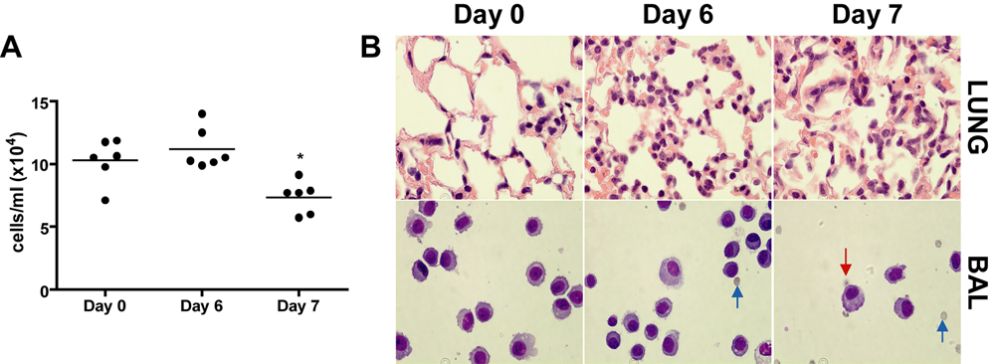
Cellular content of BALF and histopathological analysis of lung tissue from PbA-infected C57BL/6 mice. To further characterize lung injury in this model, both lung histopathology and BAL cell counts were examined during PbA infection. A. Alveolar cell counts (derived from BAL, n = 6 per group/experiment) do not differ significantly from baseline, and no neutrophil infiltration was observed. However, there is a significant decrease in BALF cellular content at Day 7 compared to Day 6 (Kruskal-Wallis test with Dunn's multiple comparison test; * p<0.05). B. H&E stained sections of lung tissue show increased inflammatory cells in the lungs of PbA infected mice, although cells appear to accumulate in the interstitium and do not accumulate in the alveolar spaces (top panel: lung). Giemsa-stained cytospin preparations of BALF cells (bottom panel: BAL) show few differences in alveolar cell types overall. Individual animals showed increased red blood cell numbers in the BALF at day 6 and 7 (blue arrows), some of which were parasitized erythrocytes (red arrow). Therefore, interstitial inflammation, without transmigration of inflammatory cells into the alveolar spaces, occurs in PbA-induced ALI.

To further characterize PbA-induced ALI, both alveolar cell counts and lung histology were examined for pathological changes. No cellular infiltration into the alveoli occurred over the course of PbA infection, but rather BALF cell counts were decreased at day 7 compared to day 6 (Figure 3A, Kruskal-Wallis test with Dunn's multiple comparison test: p<0.05). Histopathological analysis revealed interstitial pulmonary inflammation at day 6 post-infection, with increased numbers of inflammatory cells in the alveolar septae (Figure 3B, upper panel). However, consistent with the BALF cell count data, the alveolar spaces were free of inflammatory cells. By day 7 post-infection the lungs remained inflamed, with no alveolar cellular infiltration. However, lung architecture was lost in some areas and interstitial hemorrhages were seen in individual animals (Figure 3B, upper panel). Additionally, microscopic analysis of BAL cells showed few changes in cell type (Figure 3B, lower panel). Interestingly, erythrocytes, and rarely PEs, could be found in the alveoli of PbA-infected mice. To summarize, these data indicate that ALI occurs in the PbA model of severe malaria, characterized by pulmonary edema and interstitial inflammation initiated via an “inside-out” mechanism that fails to induce transmigration of inflammatory cells to the alveolar spaces.

### Genes associated with defense and immune response gene ontology (GO) categories are up-regulated in PbA-induced ALI

To examine mechanisms underlying the pathophysiology of PbA-induced ALI, expression microarray analysis of mouse lung tissue was performed. Three hundred and eighty differentially expressed genes were identified in the lungs of PbA infected C57BL/6 mice at day 6, compared to uninfected controls, at a false discovery rate of 1% using Exploratory Differential Gene Expression (EDGE) analysis [32]. Functional analysis of the differentially up-regulated genes revealed significant enrichment in the gene ontology (GO) categories of host defense and immune response, response to stress, and ribosomal activity, whereas down-regulated genes were enriched in metabolism pathways and ATPase activity (Table 1).

**Table 1:** GO categories over-represented in the differentially expressed genes from the PbA model of ALI

Enriched GO categories in up-regulated genes (FDR<0.001)
Response to biotic stimulus
Defense response
Immune response
Ribosome
Response to stress
Regulation of translation
Ribonucleoprotein complex
**Enriched GO categories in down-regulated genes (FDR<0.001)**
Metabolism
ATPase activity

**Figure 4:**
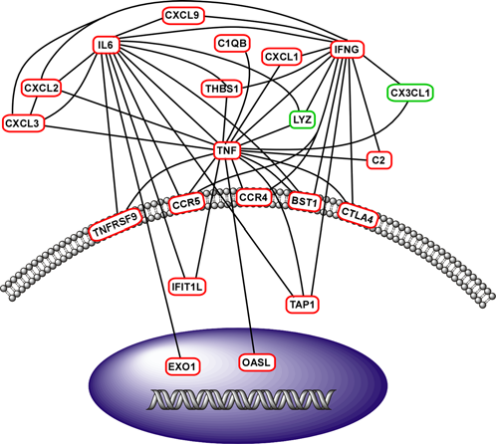
Gene network analysis identifies putative mediators of PbA-induced ALI. A gene-gene interaction network of differentially expressed lung homogenate cytokines and defense/immune response genes in the PbA infection model was created from known gene product interaction databases (Ingenuity, Adriadne and Human Protein Reference Database). Red circles denote upregulated genes and green downregulated genes. Nodes with high interconnectivity, including IFN-γ, TNF and IL-6, may be important mediators of PbA-induced ALI.

Because defense and immune response GO categories were highly enriched in the PbA model of ALI, differentially expressed genes within this functional category were further explored using network analysis. In addition to the differentially expressed genes identified using the EDGE analysis, cytokines significantly increased in lung homogenate (MIP2, IL-6, KC, and IFN-γ; Figure 2) were included in the analysis, for a total of 27 gene products. The resultant gene-gene interaction network (or interactome), created from previously identified gene product interactions, consisted of 21 genes (Figure 4). Many of these genes were up-regulated cytokines and chemokines. Additionally, the structure of this interactome was dependent upon three hubs, or nodes of high interconnectivity: namely, IFN-γ (NM_008337), TNF (NM_013693) and IL-6 (NM_031168).

### Susceptibility to ALI in PbA infection does not correlate with cerebral malaria susceptibility

**Figure 5:**
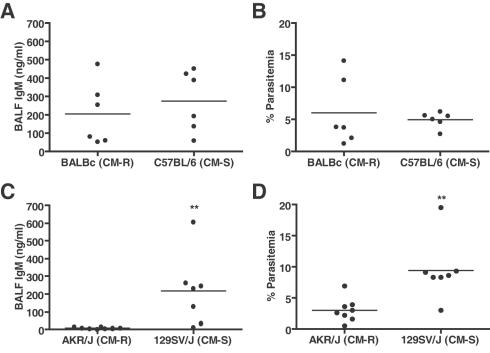
PbA-induced ALI does not correlate with CM susceptibility in in-bred mouse strains. Two pairs of CM-resistant and CM-susceptible mouse strains were infected with PbA to examine whether genetic susceptibility to CM in the PbA model parallels susceptibility to ALI. A. Despite different infection outcome, C57BL/6 (CM-susceptible, n = 6) and BALB/c (CM-resistant, n = 6) mice have equivalent BALF IgM day 6 (horizontal lines represent mean), increased over uninfected controls, and also have equivalent parasitemia (B; geometric means C57BL/6 = 4.78 and BALBc = 4.21). C. Conversely, 129SV/J (CM-susceptible, n = 7) mice develop significantly higher levels of BALF IgM (horizontal line represents mean, 2-tailed t-test, p = 0.0104) and also show significantly higher parasitemia than AKR/J (CM-resistant, n = 8) mice (D; horizontal line represents geometric mean, Mann-Whitney U test p = 0.0022). Both data sets are representative of two independent experiments. These results indicate that genetic susceptibility to CM does not correspond to ALI.

To investigate whether genetic determinants regulating susceptibility to CM in the PbA model correlate with ALI, the responses of two pairs of CM-resistant and CM-susceptible in-bred mouse strains infected with PbA were compared. At day 6 post-infection, despite their divergent outcome, C57BL/6 (CM-susceptible) and BALB/c (CM-resistant mice) have equivalently elevated BALF IgM concentrations (Figure 5A) and parasitemia (Figure 5B), although this study is limited by its small sample size (N = 6). However, CM-hyper-susceptible 129SV/J mice developed significantly higher BALF IgM levels than CM-resistant AKRJ mice (Figure 5C, Mann-Whitney test: p = 0.0012). This coincided with the 129SV/J developing significantly higher parasitemia than the AKRJ mice (Figure 5D, Mann-Whitney U test p = 0.0022). Therefore, in this model, genetic resistance to CM for example in BALB/c mice does not necessarily confer resistance to ALI.

### ALI in PbA infection is correlated with peripheral parasite burden

**Figure 6:**
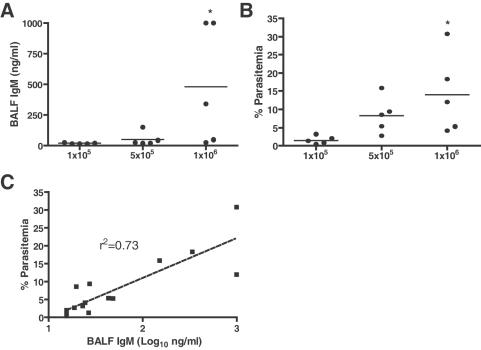
Parasite burden affects the development of ALI in PbA infection. To explore whether ALI was related to parasite burden, groups of C57BL/6 mice were inoculated with different doses of PbA, inducing a gradient of parasitemias within the same genetic background (n = 6 mice/dose (group)/experiment, representative of two independent experiments). A. BALF IgM was significantly increased at day 6 post-infection in C57BL/6 mice that received an inoculum of 1×10^6^ PE compared to those receiving a dose of 1×10^5^ (Kruskal-Wallis test with Dunn's multiple comparison test, *1×10^6^ vs. 1×10^5^ PE: p<0.05). B. An increased inoculum also corresponded with increased peripheral parasitemia at day 6 (Kruskal-Wallis test with Dunn's multiple comparison test, *1×10^6^ vs. 1×10^5^ PE: p<0.05). C. Parasitemia was positively correlated with log values of BALF IgM concentration (r^2^ = 0.73). These findings indicate that parasite burden influences the development of ALI.

Since no association was found between the development of CM and ALI, the effect of parasite burden on the development of ALI was examined. Mice with higher parasitemias were more likely to show correspondingly high levels of IgM and total protein in the BALF (Table 2). This observation led to the hypothesis that the extent of lung injury may be influenced by peripheral parasitemia, which likely reflects local parasite burden in the lung. Because diverse genetic factors influence infection in the different in-bred strains, this question was addressed using escalating parasite inocula in order to induce a spectrum of parasitemia in ALI-susceptible C57BL/6. Consistent with this hypothesis, mice that received a higher inoculum of PbA had increased concentrations of BALF IgM at day 6 post-infection (Figure 6A; Kruskal-Wallis test with Dunn's multiple comparison test, 1×10^6^ vs. 1×10^5^ PE: p<0.05) corresponding with elevated circulating parasitemias (Figure 6B; Kruskal-Wallis test with Dunn's multiple comparison test, 1×10^6^ vs. 1×10^5^ PE: p<0.05). Parasitemia was positively correlated with BALF IgM log concentration (Figure 6C; r^2^ = 0.73). These findings suggest that ALI is influenced by parasite burden and that increasing levels of circulating infected erythrocytes result in increasing levels of ALI.

**Table 2:** Summary of the correlation between peripheral parasitemia and BAL IgM or total protein levels in all in-bred strains tested at Day 6 post-infection (*p<0.05)

Strain (Sex)	ALI Susceptible	CM Susceptible	%Parasitemia (Mean±SD)	Correlation %Parasitemia vs. [IgM]	Correlation %Parasitemia vs. [Protein]
C57Bl/6 (F)	Yes	Yes	3.00±0.67	0.41	0.75*
C57Bl/6 (M)	Yes	Yes	4.93±1.22	0.42	0.66
BALB/c (M)	Yes	**No**	6.00±5.29	0.60	0.88*
129SV/J (M)	Yes	Yes	9.44±2.37	0.90*	0.86*
AKR/J (M)	**No**	**No**	3.04±2.09	−0.01	−0.45

### ALI induced by PbA infection is attenuated in *Cd36^−/−^* mice

A high peripheral parasite burden may not only stimulate proinflammatory processes but also increase the number of parasites available for sequestration in vital organs, including the lung. *P. berghei* parasites preferentially bind in the lungs of infected mice in a CD36-dependent manner (CD36; NP_031669) [27]. Given their reduced lung parasite burden, we hypothesized that *Cd36^−/−^* (*Cd36*; NM_007643) mice would be expected to be protected from ALI caused by *P. berghei* infection.

Additionally, CD36 is a receptor for thrombospondin-1 (Thbs-1; NP_035710), which was identified in the defense response interactome (Figure 4). *Thbs-1* (NM_011580) was up-regulated in the lungs of mice at day 6 post infection, compared to uninfected animals. This observation was confirmed using quantitative real-time RT-PCR analysis of an independent PbA infection (mean normalized copy number±standard deviation: Day 6 1193.3±199.2, Day 0 545.6±11.9, 2-tailed t-test with welch's correction p<0.03).

**Figure 7:**
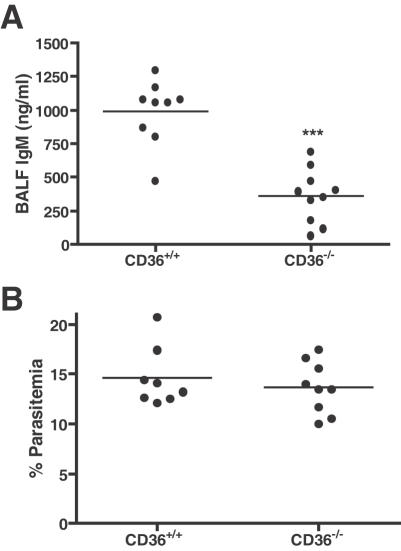
*Cd36^−/−^* mice develop significantly less ALI than wild-type mice. Previous studies have demonstrated that *P. berghei* binds in a CD36-dependent manner and sequesters preferentially in the lungs of infected mice. Hypothetically, mice with reduced lung parasite burden would be protected from the development of ALI. A. Infected *Cd36^−/−^* mice showed decreased BALF IgM concentration at Day 7 post infection compared to wildtype controls (2-tailed t-test, ***p<0.0001); however, B. *Cd36^+/+^* and *Cd36^−/−^*mice developed equivalent peripheral parasitemias and died from CM during the same time frame (data not shown). Nine CD36^−/−^ mice and 10 *Cd36^+/+^* mice were used per group, and the data are representative of two independent experiments. Therefore, ALI in this model is CD36-dependent.

Similar to previously published work, PbA-infected *Cd36^−/−^* mice were not protected from death secondary to CM (data not shown). However, *Cd36^−/−^* mice developed significantly less ALI during PbA infection compared to their wild type counterparts, as measured by BALF IgM concentration (Figure 7A, 2-tailed t-test, p<0.0001), despite having an equivalent parasitemia (Figure 7B). In summary, ALI induced by experimental murine malaria was CD36-dependent.

## Discussion

This study provides a detailed analysis of ALI that occurs in experimental murine malaria, which may provide an informative tool to study ALI and ARDS associated with human malaria infection. Mice infected with PbA develop septal inflammation and disruption of the alveolar-capillary membrane barrier, leading to a proteinaceous non-cardiogenic pulmonary edema, dependent on parasite burden and CD36. Interestingly, susceptibility to ALI does not necessarily correlate with CM development in genetically in-bred mouse strains. While all CM-susceptible strains tested developed ALI, there was differential susceptibility of CM-resistant strains to ALI, for example BALB/c develop ALI whereas others did not (AKR/J). These data suggest that ALI occurs via a mechanism distinct from the pathogenesis of CM in the PbA model.

ALI in experimental murine PbA malaria may, at least partially, represent a clinically relevant model of ALI seen in individuals with severe human malaria, since both share similar histopathology features, parasite sequestration in the lung capillaries and alveolar-capillary membrane barrier disruption leading to pulmonary edema. Lung histology from both PbA and *P. falciparum* infections shows an edematous interstitium with leukocyte infiltration [13]. PEs and leukocytes sequester in the pulmonary microvasculature in human malaria infections, as demonstrated by both ultrastructural studies [13],[14],[15] and a reduced pulmonary capillary vascular component volume [2],[12]. Additionally, hemorrhage is a classic feature of non-malarial human ALI/ARDS [29],[33] and histopathological reports on malaria-induced ALI indicate that focal alveolar hemorrhages occur in humans [34],[35], similar to those observed in the PbA model. Progressive alveolar-capillary dysfunction has been reported in individuals with malaria immediately following appropriate antimicrobial therapy [2],[12]. This post-treatment lung damage has been attributed to the host inflammatory response, and it appears that pulmonary complications in human malaria result from a combination of PE sequestration, and the corresponding host inflammatory response to parasite burden. As in human malaria, ALI in the PbA model is partially mediated by parasite burden and sequestration (Figures 5 & 6), but also likely occurs as a response to parasite-driven inflammatory responses. Indeed, work using an anti-*P. falciparum* GPI vaccine in the PbA model showed markedly reduced pulmonary edema in immunized versus sham-immunized animals [26].

As with any animal model of disease, there are limitations to the correlations that can be drawn to human disease, especially since limited studies have examined ALI in human malaria infection. It is not possible to comment on how the BAL findings from this model relate to human malaria, because these studies have not been performed and obtaining BAL samples from severe malaria patients, especially in a field setting, may present challenges. Additionally, while certain in-bred mouse strains show differential susceptibility to PbA-induced ALI and CM–BALB/c mice develop ALI but are resistant to CM–this may not necessarily reflect what occurs in human malaria. Although case reports and other studies have demonstrated that respiratory involvement and ALI can occur in non-cerebral malaria [1],[2],[4],[12], *o*ther studies have shown that lung parasite burden parallels that in the brain [34] and that ALI commonly occurs in conjunction with CM [3],[8],[34],[35].

This study examined transcriptional profiles from the lungs of PbA infected mice. The defense/immune response interactome (Figure 4) is structured around three hubs of high interconnectivity–TNF, IFN-γ and IL-6, all of which play significant roles in host pro-inflammatory responses to malaria [36],[37],[38]. The functional stability of genetic networks is highly dependent on nodes of high interactivity [39], indicating they may play a pivotal role in the transcriptional response to PbA-induced ALI and thus may be promising therapeutic targets. TNF has been well-established as a key mediator of ALI [40],[41],[42], which is also likely the case in PbA, since TNF levels were significantly increased in the lung tissue of infected mice. However, since IFN-γ, TNF and IL-6 are involved in multiple biological processes in PbA infection, it may be possible to more effectively modulate these key hubs by targeting a molecule or pathway shared by them, such as THBS-1 (Figure 4). THBS-1 was of particular interest among the interactome because it binds and signals primarily via CD36 (NP_000063) [43],[44]. THBS-1 (NP_003237) has also been identified as an cellular receptor for PE adhesion [45], and additionally, soluble THBS-1 binds to PEs, augmenting adhesion to endothelial cells via CD36 under physiological flow conditions [46],[47]. The upregulation of *thbs-1* expression could play an important role in PbA-induced ALI by increasing CD36-mediated cytoadherance in the lung, especially since CD36 is a primary receptor for PEs in the pulmonary vascular endothelium [27]. Although CD36 deficiency does not affect CM development and survival in the PbA model [27], our work demonstrates that *Cd36^−/−^* mice develop significantly less ALI compared to wild-type controls. This finding suggests that malaria-induced ALI occurs via a CD36-dependent pathogenic mechanism.

These findings may appear to conflict with previous work by our group, which has argued that CD36 may be beneficial in the immune response to malaria via its role as a receptor for non-opsonic phagocytosis of PEs by macrophages [48],[49],[50],[51]. Recent studies, using chimeric mice expressing CD36 only on hematopoietic cells, showed that CD36 on myeloid cells (i.e. the hematopoeitic compartment) but not on endothelial cells (the non-hematopoeitic compartment) conferred protection to CM in the PbA model [52]. However, there is conflicting evidence that PEs that bind CD36 are associated with severe disease. Parasites isolated from severe malaria patients in Thailand were shown to preferentially bind ICAM-1 on lung endothelium *in vitro* compared to those from uncomplicated patients [53]. Similarly among children with severe malaria in Africa, parasite binding to CD36 was inversely related to disease severity [54], but another study found that CD36 binding was equivalent between parasitized erythrocytes derived from CM patients or community controls [55]. However none of these studies specifically examined parasite isolates from patients displaying symptoms of ALI.

Additionally, polymorphisms in CD36, and CD36 deficiency, exist as natural variants in malaria endemic regions, including Asia [56] and Africa [57]. CD36 polymorphisms have been associated with both increased [58] and decreased susceptibility to CM [59]. Moreover, a specific non-sense mutation in CD36 was shown to be significantly associated with protection from respiratory distress in African children [60]. These studies examined different polymorphisms, which may reflect differential protein function or expression in different cell types. If CD36 mutations do confer susceptibility to cerebral malaria [53], these mutations may be maintained in human populations through selection pressure of another prevalent infection other than malaria, or perhaps even by resistance to malaria-associated ALI. Additional studies are required to clarify the association between CD36 polymorphisms and severe malaria including ALI. Taken together, the available data suggest a dual role for CD36 in malaria infection. Specifically, as a pattern recognition receptor on myeloid cell lineages, CD36 may contribute to innate immune response and parasite clearance but at high parasite density, endothelial cell CD36 may also play a role, at least in the mouse model, in the development of tissue injury at sites such as the lung.

## Methods

### Mice and parasites

Animal use protocols were reviewed and approved by the Faculty of Medicine Advisory Committee on Animal Services at the University of Toronto and all experiments were conducted according to the animal ethics guidelines of the University of Toronto. C57Bl/6 and BALB/c mice were obtained from Charles River Laboratories (Senneville QC), and 129SV/J and AKR/J were purchased from Jackson Laboratories (Bar Harbor ME). CD36^−/−^ mice (on a C57Bl/6 background, a gift from Maria Febbraio (New York NY)) were bred and maintained at the University of Toronto animal facility. Mice were 8–12 weeks of age and groups were matched by sex. Each experiment was performed twice, with 6–10 mice per group, as outlined in individual figure legends.

Cryopreserved PbA (MR4, Vannassas MA) was thawed and passaged through naïve C57Bl/6 donor mice until parasitemia in the passage animals reached approximately 10%. On day 0, experimental mice were infected by intraperitoneal injection with freshly isolated PbA. Male mice were inoculated with 5×10^5^ PE and females with 1×10^6^ PE, inocula that reproducibly show 100% mortality in C57BL/6 mice. Parasitemia was monitored daily after Day 3 using thin blood smears stained with modified Giemsa (Protocol Hema 3 Stain Set; Sigma, Oakville ON).

### Bronchoalveolar lavage fluid (BALF) analysis

At Day 6 or 7, infected mice and uninfected controls were euthanized using isofluorane and BALF of both lungs was obtained by instillation and aspiration of three 0.5 ml aliquots of Dubecco's Phosphate Buffered Saline (PBS; Gibco/Invitrogen, Burlington ON) [33]. The BALF was spun at 800×g at 4°C for 5 min, and the supernatant was removed and stored at −80°C for further protein analysis. The cell pellet was resuspended in 1 ml ice-cold PBS. Total cell numbers were determined using a hemocytometer and differential cell counts were determined by cytocentrifugation and modified Giemsa staining. BALF concentrations of MIP-2, mouse keratinocyte-derived cytokine (KC or IL-8), IL-1α (NP_034684), IL-6, IL-10, TNF-α , (that measures TNF and LT-α) and IFN-γ were determined by multiplex immunoassay (Luminex 100) using cytokine-specific bead kits according to the manufacturer's protocols (R&D Systems, Minneapolis MN). TNF-α levels in lung homogenates were confirmed using a standard sandwich ELISA according to the manufacturer's protocol (eBioscience, San Diego CA). BALF total protein concentration was measured using a BCA protein assay (Sigma), and BALF IgM concentration was determined by ELISA (Bethyl Laboratories, Montgomery TX).

### Lung homogenate analysis

Lungs were excised, weighed and homogenized in 2ml PBS/0.5g lung tissue for 30 sec. using a ULTRA -TURRAX® disperser (IKA, Wilmington NC). Homogenates were stored at −80°C for further cytokine analysis. Cytokine concentrations were measured as described above.

### Lung histology

Lungs were fixed for histology at 20cm H_2_O with 4% paraformaldehyde buffered in PBS. After fixation, the lungs were embedded in paraffin, cut into 4-µm sections, and stained with hematoxylin and eosin (H&E).

### RNA isolation and microarray hybridization

Lungs were excised immediately following euthanasia, snap-frozen in liquid nitrogen and stored at −80°C until use. Total RNA was extracted using Trizol reagent (Invitrogen) according to the manufacturer's instructions, and mRNA was purified using an Oligo-dT cellulose column (NEB, Mississauga, ON) as described previously [61]. cDNA with incorporated 5-(3-aminoallyl)-2′deoxyuridine-5′-triphosphate (AAdUTP; Sigma, Oakville ON) was reverse-transcribed from 1–2 µg mRNA. Purified cDNA was coupled with N-hydroxysuccinimide esters of Cy3 or Cy5 (GE Lifesciences, Baie d'Urfe QC). Cy3 and Cy5-labeled cDNA pairs and Agilent control spots were added to a final volume of 0.5ml hybridization buffer (1 M NaCl, 0.5% sodium sarcosine, 50 mM methyl ethane sulfonate (MES), pH 6.5, 33% formamide and 40 µg salmon sperm DNA (Invitrogen)). Hybridizations were performed in Agilent hybridization (Agilent, Palo Alto CA) chambers at 42°C with rotation for 18–24 hours. Slides were washed in 6×SSPE, 0.005% sarcosine, followed by 0.06×SSPE, allowed to dry and scanned with a 4000A microarray scanner (Axon Instruments, Union City CA).

TIFF images were quantified with GenePix (Axon Instruments). Variance stabilizing normalization [62] and loess smoothing were applied in Bioconductor [63] and the data were transformed to log2 scale.

Each array was hybridized with cDNA transcribed from an RNA pool of 5 C57BL/6 mice per timepoint (Day 0 and 6) and technical replicates (dye-swap) experiments were performed for both time points.

### Microarray Data Analysis

The PbA ALI dataset (GSE9497) and mouse/PbA microarray platform (GPL4220) were deposited in the GEO database (www.ncbi.nlm.nih.gov/projects/geo/) in accordance with MIAME guidelines.

Probe mapping was performed as previously described [28] and a total of 9724 unique mouse genes, annotated using the Entrez Gene database, were included in the analysis. Since RNA was pooled from whole lung homogenates and replication was limited to dye-switching experiments, a statistical framework developed for the analysis of single cDNA microarray experiments–Exploratory Differential Gene Expression (EDGE)–was utilized [32]. This program was implemented in the R software environment (www.r-project.org) to determine statistical significance in each microarray experiment. The problem of multiple hypothesis testing was addressed using false discovery analysis based on *Q*-values [64]. A gene was deemed significantly differentially expressed if its *Q*-value was ≤0.01 in at least one of the dye-switching experiments and the direction of change (i.e., up or down-regulation relative to uninfected controls) was consistent in both experiments.

### Gene Ontology (GO) Analysis

Functional annotation of the genes was obtained from Gene Ontology Consortium's database [65], based on their respective molecular function, biological process, or cellular component. Enriched functional categories within differentially expressed genes were determined using the Expression Analysis Systematic Explorer (EASE) algorithm [66]. A variant of the one-tailed Fisher exact probability test based on the hypergeometric distribution was used to calculate *P*-values. Generated *P*-values indicated whether a given GO process is over-represented compared to what would be expected by random sampling. Multiple hypothesis testing was addressed by performing permutation analysis (n = 1000) and selecting a false discovery rate cutoff of ≤0.001.

### Gene Network Analysis

A gene-gene interaction network was created by mining gene product interactions from the following databases: Ingenuity [67], Adriadne [68], and Human Protein Reference Database [69]. These knowledge bases have been manually and computationally compiled through extensive literature searches. Molecular relationships consisting of direct physical, transcriptional, and enzymatic interactions among gene products serve as the basis for creating genetic networks from gene or protein expression data.

### Quantitative real-time RT-PCR

cDNA was synthesized from 0.5 µg of mRNA using Superscript II reverse transcriptase with Oligo (dT)_12-18_ primers (Invitrogen). Serial dilutions of mouse genomic DNA were used as standards [70]. gDNA standards or cDNA were added to the qPCR reaction containing 1× Power Sybr Green Master Mix (Applied Biosystems) and 0.5 µM primers in a final volume of 10 µl. qPCR was performed using the ABI Prism^®^ 7900HT Sequence Detection System (Applied Biosystems). Copy numbers were normalized to 3 mouse housekeeping genes–*Hprt*, *Sdha*, and *Ywhaz* [71]. Forward (fwd) and reverse (rvs) primer sequences are as follows: Thbs1-fwd: TGT GGA CTT CAG CGG TAC CTT CTT; Thbs1-rvs: GGA CTG GGT GAC TTG TTT CCA CAT; Hprt-fwd: GGAGTCCTGTTGATGTTGCCAGTA, Hprt-rvs: GGGACGCAGCAACTGACATTTCTA; Sdha-fwd: TCACGTCTACCTGCAGTTGCATCA, Sdha-rvs: TGACATCCACACCAGCGAAGATCA; Ywhaz-fwd: AGCAGGCAGAGCGATATGATGACA, Ywhaz-rvs: TCCCTGCTCAGTGACAGACTTCAT.
